# Supplementary material for: The Burden of Gastric Cancer Attributable to High Sodium Intake: A Longitudinal Study from 1990 to 2019 in China
Source: Nutrients. 2023 Dec 13;15(24):5088. doi: 10.3390/nu15245088 (PMC10745903; doi:10.3390/nu15245088)
Supplement: Supplementary file 1 [file nutrients-15-05088-s001.zip › Supplementary Tables 1.pdf]

Supplementary Table S1. Deaths and ASMR of gastric cancer disease attributable-high sodium intake in 1990 and 2019 and the temporal trends from 1990-2019.

Characteristics	1990		2019			1990-2019
	Deaths cases, No. (95% UI)	ASMR per 100,000 No.(95% UI)	Deaths cases, No. (95% UI)	ASMR per 100,000 No. (95% UI)	PAFs % (95% UI)	EAPC(%) in ASMR No. (95% CI)
**Region**						
Anhui	2146.88 (46.72 to 7905.63)	5.49 (0.12 to 20.37)	2620.63 (55.77 to 9898)	2.87 (0.06 to 10.85)	8.89 (0.21 to 33.04)	-2.03 (-2.44 to -1.63)
Beijing	174.73 (3.86 to 651.83)	1.98 (0.04 to 7.31)	252.11 (5.41 to 921.33)	0.78 (0.02 to 2.85)	8.90 (0.21 to 32.91)	-3.32 (-3.41 to -3.23)
Chongqing	278.69 (6.04 to 1032.87)	2.36 (0.05 to 8.71)	643.60 (13.83 to 2461.93)	1.52 (0.03 to 5.85)	8.69 (0.21 to 32.50)	-1.14 (-1.54 to -0.74)
Fujian	1042.99 (22.75 to 3940.85)	5.22 (0.12 to 19.57)	1234.75 (26.77 to 4643.99)	2.47 (0.05 to 9.31)	8.81(0.21 to 32.80)	-2.63 (-2.72 to -2.54)
Gansu	612.58 (14.23 to 2391.08)	4.46 (0.11 to 17.82)	975.81 (22.56 to 3759.51)	2.84 (0.07 to 10.99)	8.04 (0.21 to 31.41)	-1.44 (-1.89 to -0.98)
Guangdong	890.53 (19.77 to 3437.91)	1.98 (0.04 to 7.65)	1215.51 (27.48 to 4638.79)	0.93 (0.02 to 3.55)	8.59 (0.21 to 32.40)	-2.23 (-2.68 to -1.77)
Guangxi	621.23 (13.68 to 2397.13)	2.17 (0.05 to 8.51)	1003.72 (22.01 to 3781.63)	1.63 (0.04 to 6.17)	8.46 (0.21 to 32.10)	-0.38 (-0.83 to 0.07)
Guizhou	413.55 (9.05 to 1525.41)	2.03 (0.05 to 7.56)	672.22 (14.67 to 2516.46)	1.57 (0.03 to 5.96)	8.68 (0.21 to 32.59)	-0.38 (-0.71 to -0.05)
Hainan	160.62 (3.58 to 615.73)	3.60 (0.08 to 13.95)	256.92 (5.75 to 989.52)	2.30 (0.05 to 8.89)	8.42 (0.21 to 32.14)	-1.12 (-1.42 to -0.82)
Hebei	1509.07 (33.34 to 5589.76)	3.41 (0.08 to 12.69)	2080.59 (44.65 to 7700.39)	2.04 (0.04 to 7.56)	8.83 (0.21 to 32.91)	-1.63 (-2.09 to -1.18)
Heilongjiang	589.98 (13.10 to 2252.24)	2.98 (0.07 to 11.43)	929.05 (20.52 to 3481.43)	1.57 (0.04 to 6.02)	8.52 (0.21 to 32.26)	-1.76 (-2.05 to -1.46)

Characteristics	1990		2019			1990-2019
	Deaths cases, No. (95% UI)	ASMR per 100,000 No.(95% UI)	Deaths cases, No. (95% UI)	ASMR per 100,000 No. (95% UI)	PAFs % (95% UI)	EAPC(%) in ASMR No. (95% CI)
Henan	2611.86 (57.8 to 9561.77)	4.28 (0.10 to 15.83)	2950.71 (64.73 to 10986.83)	2.41 (0.05 to 9.02)	8.79 (0.21 to 32.91)	-1.91 (-2.30 to -1.52)
Hong Kong	81.96 (1.92 to 299.89)	1.49 (0.03 to 5.46)	107.24 (2.29 to 409.98)	0.72 (0.02 to 2.72)	8.79 (0.21 to 32.88)	-2.46 (-2.69 to -2.22)
Hubei	1035.70 (23.33 to 3810.60)	2.77 (0.06 to 10.18)	1492.15 (32.15 to 5669.98)	1.73 (0.04 to 6.54)	8.72 (0.21 to 32.71)	-1.06 (-1.44 to -0.68)
Hunan	762.78 (17.22 to 2820.83)	1.75 (0.04 to 6.47)	1132.43 (24.55 to 4254.50)	1.16 (0.03 to 4.36)	8.75 (0.21 to 32.69)	-0.96 (-1.25 to -0.66)
Inner Mongolia	357.90 (7.78 to 1336.06)	2.86 (0.06 to 10.7)	574.57 (12.24 to 2104.58)	1.69 (0.04 to 6.19)	8.88 (0.21 to 32.98)	-1.73 (-2.08 to -1.38)
Jiangsu	2330.12 (52.07 to 8597.48)	4.34 (0.10 to 16.1)	3034.31 (65.72 to 11195.55)	2.22 (0.05 to 8.17)	8.82 (0.21 to 32.89)	-2.14 (-2.65 to -1.62)
Jiangxi	846.71 (18.73 to 3142.73)	3.45 (0.08 to 12.83)	1016.95 (21.67 to 3815.86)	1.80 (0.04 to 6.80)	8.89 (0.21 to 33.01)	-2.01 (-2.25 to -1.77)
Jilin	468.81 (10.31 to 1808.05)	3.02 (0.07 to 11.66)	530.16 (11.84 to 1939.54)	1.25 (0.03 to 4.65)	8.75 (0.21 to 32.75)	-3.19 (-3.62 to -2.75)
Liaoning	859.80 (19.15 to 3286.72)	3.08 (0.07 to 11.86)	1355.75 (29.84 to 5145.03)	1.76 (0.04 to 6.73)	8.59 (0.21 to 32.39)	-1.55 (-1.8 to -1.31)
Macao	4.15 (0.09 to 15.17)	1.52 (0.03 to 5.52)	5.83 (0.12 to 22.49)	0.63 (0.01 to 2.42)	8.83 (0.21 to 32.97)	-3.14 (-3.34 to -2.95)
Ningxia	113.15 (2.47 to 438.24)	4.82 (0.11 to 18.84)	243.82 (5.48 to 937.69)	3.41 (0.08 to 13.20)	8.44 (0.21 to 32.27)	-0.98 (-1.13 to -0.82)
Qinghai	127.81 (2.69 to 476.30)	5.44 (0.12 to 20.04)	273.51 (5.83 to 1019.98)	4.36 (0.10 to 16.29)	8.88 (0.21 to 32.97)	-0.51 (-0.71 to -0.3)
Shaanxi	796.28 (17.31 to 2954.11)	3.61 (0.08 to 13.51)	1044.35 (22.34 to 3865.56)	1.98 (0.04 to 7.39)	8.84 (0.21 to 32.83)	-1.94 (-2.31 to -1.58)

Characteristics	1990		2019			1990-2019
	**Deaths cases, No. (95% UI)**	**ASMR per 100,000 No.(95% UI)**	**Deaths cases, No. (95% UI)**	**ASMR per 100,000 No. (95% UI)**	**PAFs % (95% UI)**	**EAPC(%) in ASMR No. (95% CI)**
Shandong	2363.43 (51.86 to 8795.77)	3.73 (0.08 to 14.01)	3543.69 (78.12 to 13024.72)	2.31 (0.05 to 8.52)	8.86 (0.21 to 32.97)	-1.35 (-1.88 to -0.82)
Shanghai	492.02 (10.58 to 1852.30)	3.50 (0.08 to 13.27)	566.30 (12.04 to 2139.24)	1.34 (0.03 to 5.06)	8.70 (0.21 to 32.66)	-3.34 (-3.43 to -3.25)
Shanxi	926.59 (20.74 to 3533.39)	4.64 (0.11 to 17.87)	1494.43 (32.86 to 5639.88)	3.16 (0.07 to 12.10)	8.59 (0.21 to 32.38)	-1.05 (-1.52 to -0.57)
Sichuan	2491.59 (54.53 to 9295.86)	3.11 (0.07 to 11.65)	2747.61 (59.9 to 10112.62)	2.09 (0.05 to 7.76)	8.70 (0.21 to 32.57)	-1.14(-1.91 to -0.36)
Tianjin	144.33 (3.25 to 539.00)	2.04 (0.05 to 7.69)	199.11 (4.28 to 773.61)	0.94 (0.02 to 3.63)	8.60 (0.21 to 32.48)	-2.49 (-2.67 to -2.32)
Tibet	60.52 (1.32 to 228.81)	4.12 (0.09 to 15.49)	62.28 (1.31 to 227.56)	2.35 (0.05 to 8.60)	8.92 (0.21 to 32.95)	-2.13 (-2.37 to -1.89)
Xinjiang	269.27 (5.72 to 1019.43)	3.19 (0.07 to 12.12)	559.31 (11.97 to 2077.37)	2.34 (0.05 to 8.76)	8.88 (0.21 to 32.84)	-0.75 (-0.96 to -0.54)
Yunnan	385.05 (8.44 to 1452.29)	1.59 (0.04 to 6.00)	677.78 (14.8 to 2498.95)	1.24 (0.03 to 4.61)	8.71 (0.21 to 32.70)	-0.36 (-0.69 to -0.02)
Zhejiang	1255.84 (27 to 4654.13)	3.68 (0.08 to 13.67)	1634.26 (34.75 to 5953.92)	1.85 (0.04 to 6.76)	9.00 (0.21 to 33.13)	-2.14 (-2.44 to -1.83)

ASMR=age-standardized mortality rate; PAF=population attributable fraction; EAPC=estimated annual percentage change.
