# Supplementary material for: The Burden of Gastric Cancer Attributable to High Sodium Intake: A Longitudinal Study from 1990 to 2019 in China
Source: Nutrients. 2023 Dec 13;15(24):5088. doi: 10.3390/nu15245088 (PMC10745903; doi:10.3390/nu15245088)
Supplement: Supplementary file 1 [file nutrients-15-05088-s001.zip › Supplementary Tables 2.pdf]

Supplementary Table S2. DALYs and ASDR of gastric cancer disease attributable to high sodium intake in 1990 and 2019 and the temporal trends from 1990 to 2019.

Characteristics	1990		2019			1990-2019
	**Deaths cases, No. (95% UI)**	**ASDR per 100,000 No.(95% UI)**	**DALYs, No. (95% UI)**	**ASDR per 100,000 No. (95% UI)**	**PAFs % (95% UI)**	**EAPC(%) in ASMR No. (95% CI)**
**Region**						
Anhui	2146.88 (46.72 to 7905.63)	5.49 (0.12 to 20.37)	2620.63 (55.77 to 9898)	2.87 (0.06 to 10.85)	8.89 (0.21 to 33.04)	-2.03 (-2.44 to -1.63)
Beijing	174.73 (3.86 to 651.83)	1.98 (0.04 to 7.31)	252.11 (5.41 to 921.33)	0.78 (0.02 to 2.85)	8.90 (0.21 to 32.91)	-3.32 (-3.41 to -3.23)
Chongqing	278.69 (6.04 to 1032.87)	2.36 (0.05 to 8.71)	643.60 (13.83 to 2461.93)	1.52 (0.03 to 5.85)	8.69 (0.21 to 32.50)	-1.14 (-1.54 to -0.74)
Fujian	1042.99 (22.75 to 3940.85)	5.22 (0.12 to 19.57)	1234.75 (26.77 to 4643.99)	2.47 (0.05 to 9.31)	8.81(0.21 to 32.80)	-2.63 (-2.72 to -2.54)
Gansu	612.58 (14.23 to 2391.08)	4.46 (0.11 to 17.82)	975.81 (22.56 to 3759.51)	2.84 (0.07 to 10.99)	8.04 (0.21 to 31.41)	-1.44 (-1.89 to -0.98)
Guangdong	890.53 (19.77 to 3437.91)	1.98 (0.04 to 7.65)	1215.51 (27.48 to 4638.79)	0.93 (0.02 to 3.55)	8.59 (0.21 to 32.40)	-2.23 (-2.68 to -1.77)
Guangxi	621.23 (13.68 to 2397.13)	2.17 (0.05 to 8.51)	1003.72 (22.01 to 3781.63)	1.63 (0.04 to 6.17)	8.46 (0.21 to 32.10)	-0.38 (-0.83 to 0.07)
Guizhou	413.55 (9.05 to 1525.41)	2.03 (0.05 to 7.56)	672.22 (14.67 to 2516.46)	1.57 (0.03 to 5.96)	8.68 (0.21 to 32.59)	-0.38 (-0.71 to -0.05)
Hainan	160.62 (3.58 to 615.73)	3.60 (0.08 to 13.95)	256.92 (5.75 to 989.52)	2.30 (0.05 to 8.89)	8.42 (0.21 to 32.14)	-1.12 (-1.42 to -0.82)
Hebei	1509.07 (33.34 to 5589.76)	3.41 (0.08 to 12.69)	2080.59 (44.65 to 7700.39)	2.04 (0.04 to 7.56)	8.83 (0.21 to 32.91)	-1.63 (-2.09 to -1.18)
Heilongjiang	589.98 (13.10 to 2252.24)	2.98 (0.07 to 11.43)	929.05 (20.52 to 3481.43)	1.57 (0.04 to 6.02)	8.52 (0.21 to 32.26)	-1.76 (-2.05 to -1.46)

Characteristics	1990		2019			1990-2019
	Deaths cases, No. (95% UI)	ASDR per 100,000 No.(95% UI)	DALYs, No. (95% UI)	ASDR per 100,000 No. (95% UI)	PAFs % (95% UI)	EAPC(%) in ASMR No. (95% CI)
Henan	2611.86 (57.8 to 9561.77)	4.28 (0.10 to 15.83)	2950.71 (64.73 to 10986.83)	2.41 (0.05 to 9.02)	8.79 (0.21 to 32.91)	-1.91 (-2.30 to -1.52)
Hong Kong	81.96 (1.92 to 299.89)	1.49 (0.03 to 5.46)	107.24 (2.29 to 409.98)	0.72 (0.02 to 2.72)	8.79 (0.21 to 32.88)	-2.46 (-2.69 to -2.22)
Hubei	1035.70 (23.33 to 3810.60)	2.77 (0.06 to 10.18)	1492.15 (32.15 to 5669.98)	1.73 (0.04 to 6.54)	8.72 (0.21 to 32.71)	-1.06 (-1.44 to -0.68)
Hunan	762.78 (17.22 to 2820.83)	1.75 (0.04 to 6.47)	1132.43 (24.55 to 4254.50)	1.16 (0.03 to 4.36)	8.75 (0.21 to 32.69)	-0.96 (-1.25 to -0.66)
Inner Mongolia	357.90 (7.78 to 1336.06)	2.86 (0.06 to 10.7)	574.57 (12.24 to 2104.58)	1.69 (0.04 to 6.19)	8.88 (0.21 to 32.98)	-1.73 (-2.08 to -1.38)
Jiangsu	2330.12 (52.07 to 8597.48)	4.34 (0.10 to 16.1)	3034.31 (65.72 to 11195.55)	2.22 (0.05 to 8.17)	8.82 (0.21 to 32.89)	-2.14 (-2.65 to -1.62)
Jiangxi	846.71 (18.73 to 3142.73)	3.45 (0.08 to 12.83)	1016.95 (21.67 to 3815.86)	1.80 (0.04 to 6.80)	8.89 (0.21 to 33.01)	-2.01 (-2.25 to -1.77)
Jilin	468.81 (10.31 to 1808.05)	3.02 (0.07 to 11.66)	530.16 (11.84 to 1939.54)	1.25 (0.03 to 4.65)	8.75 (0.21 to 32.75)	-3.19 (-3.62 to -2.75)
Liaoning	859.80 (19.15 to 3286.72)	3.08 (0.07 to 11.86)	1355.75 (29.84 to 5145.03)	1.76 (0.04 to 6.73)	8.59 (0.21 to 32.39)	-1.55 (-1.8 to -1.31)
Macao	4.15 (0.09 to 15.17)	1.52 (0.03 to 5.52)	5.83 (0.12 to 22.49)	0.63 (0.01 to 2.42)	8.83 (0.21 to 32.97)	-3.14 (-3.34 to -2.95)
Ningxia	113.15 (2.47 to 438.24)	4.82 (0.11 to 18.84)	243.82 (5.48 to 937.69)	3.41 (0.08 to 13.20)	8.44 (0.21 to 32.27)	-0.98 (-1.13 to -0.82)
Qinghai	127.81 (2.69 to 476.30)	5.44 (0.12 to 20.04)	273.51 (5.83 to 1019.98)	4.36 (0.10 to 16.29)	8.88 (0.21 to 32.97)	-0.51 (-0.71 to -0.3)
Shaanxi	796.28 (17.31 to 2954.11)	3.61 (0.08 to 13.51)	1044.35 (22.34 to 3865.56)	1.98 (0.04 to 7.39)	8.84 (0.21 to 32.83)	-1.94 (-2.31 to -1.58)

Characteristics	1990		2019			1990-2019
	Deaths cases, No. (95% UI)	ASDR per 100,000 No.(95% UI)	DALYs, No. (95% UI)	ASDR per 100,000 No. (95% UI)	PAFs % (95% UI)	EAPC(%) in ASMR No. (95% CI)
Shandong	2363.43 (51.86 to 8795.77)	3.73 (0.08 to 14.01)	3543.69 (78.12 to 13024.72)	2.31 (0.05 to 8.52)	8.86 (0.21 to 32.97)	-1.35 (-1.88 to -0.82)
Shanghai	492.02 (10.58 to 1852.30)	3.50 (0.08 to 13.27)	566.30 (12.04 to 2139.24)	1.34 (0.03 to 5.06)	8.70 (0.21 to 32.66)	-3.34 (-3.43 to -3.25)
Shanxi	926.59 (20.74 to 3533.39)	4.64 (0.11 to 17.87)	1494.43 (32.86 to 5639.88)	3.16 (0.07 to 12.10)	8.59 (0.21 to 32.38)	-1.05 (-1.52 to -0.57)
Sichuan	2491.59 (54.53 to 9295.86)	3.11 (0.07 to 11.65)	2747.61 (59.9 to 10112.62)	2.09 (0.05 to 7.76)	8.70 (0.21 to 32.57)	-1.14(-1.91 to -0.36)
Tianjin	144.33 (3.25 to 539.00)	2.04 (0.05 to 7.69)	199.11 (4.28 to 773.61)	0.94 (0.02 to 3.63)	8.60 (0.21 to 32.48)	-2.49 (-2.67 to -2.32)
Tibet	60.52 (1.32 to 228.81)	4.12 (0.09 to 15.49)	62.28 (1.31 to 227.56)	2.35 (0.05 to 8.60)	8.92 (0.21 to 32.95)	-2.13 (-2.37 to -1.89)
Xinjiang	269.27 (5.72 to 1019.43)	3.19 (0.07 to 12.12)	559.31 (11.97 to 2077.37)	2.34 (0.05 to 8.76)	8.88 (0.21 to 32.84)	-0.75 (-0.96 to -0.54)
Yunnan	385.05 (8.44 to 1452.29)	1.59 (0.04 to 6.00)	677.78 (14.8 to 2498.95)	1.24 (0.03 to 4.61)	8.71 (0.21 to 32.70)	-0.36 (-0.69 to -0.02)
Zhejiang	1255.84 (27 to 4654.13)	3.68 (0.08 to 13.67)	1634.26 (34.75 to 5953.92)	1.85 (0.04 to 6.76)	9.00 (0.21 to 33.13)	-2.14 (-2.44 to -1.83)

DALYs=disability-adjusted life years; ASDR=age-standardized DALYs rate; PAF=population attributable fraction; EAPC=estimated annual percentage change.
